# Biocompatibility of a Conjugated Polymer Retinal Prosthesis in the Domestic Pig

**DOI:** 10.3389/fbioe.2020.579141

**Published:** 2020-10-15

**Authors:** José Fernando Maya-Vetencourt, Stefano Di Marco, Maurizio Mete, Mattia Di Paolo, Domenico Ventrella, Francesca Barone, Alberto Elmi, Giovanni Manfredi, Andrea Desii, Walter G. Sannita, Silvia Bisti, Guglielmo Lanzani, Grazia Pertile, Maria Laura Bacci, Fabio Benfenati

**Affiliations:** ^1^Center for Synaptic Neuroscience and Technology, Istituto Italiano di Tecnologia, Genova, Italy; ^2^Istituto di Ricovero e Cura a Carattere Scientifico, San Martino Hospital, Genova, Italy; ^3^Department of Biology, University of Pisa, Pisa, Italy; ^4^Department of Biotechnology and Applied Clinical Science, University of L’Aquila, L’Aquila, Italy; ^5^Department of Ophthalmology, Sacro Cuore Don Calabria Hospital, Negrar, Italy; ^6^Department of Veterinary Medical Sciences, University of Bologna, Bologna, Italy; ^7^Center for Nano Science and Technology, Istituto Italiano di Tecnologia, Milan, Italy; ^8^Consorzio Interuniversitario INBB, Rome, Italy

**Keywords:** retinal degeneration, retinitis pigmentosa, biomedical pig, retinal prosthesis, conjugated polymers

## Abstract

The progressive degeneration of retinal photoreceptors is one of the most significant causes of blindness in humans. Conjugated polymers represent an attractive solution to the field of retinal prostheses, and a multi-layer fully organic prosthesis implanted subretinally in dystrophic Royal College of Surgeons (RCS) rats was able to rescue visual functions. As a step toward human translation, we report here the fabrication and *in vivo* testing of a similar device engineered to adapt to the human-like size of the eye of the domestic pig, an excellent animal paradigm to test therapeutic strategies for photoreceptors degeneration. The active conjugated polymers were layered onto two distinct passive substrates, namely electro-spun silk fibroin (ESF) and polyethylene terephthalate (PET). Naive pigs were implanted subretinally with the active device in one eye, while the contralateral eye was sham implanted with substrate only. Retinal morphology and functionality were assessed before and after surgery by means of *in vivo* optical coherence tomography and full-field electroretinogram (ff-ERG) analysis. After the sacrifice, the retina morphology and inflammatory markers were analyzed by immunohistochemistry of the excised retinas. Surprisingly, ESF-based prostheses caused a proliferative vitreoretinopathy with disappearance of the ff-ERG b-wave in the implanted eyes. In contrast, PET-based active devices did not evoke significant inflammatory responses. As expected, the subretinal implantation of both PET only and the PET-based prosthesis locally decreased the thickness of the outer nuclear layer due to local photoreceptor loss. However, while the implantation of the PET only substrate decreased the ff-ERG b-wave amplitude with respect to the pre-implant ERG, the eyes implanted with the active device fully preserved the ERG responses, indicating an active compensation of the surgery-induced photoreceptor loss. Our findings highlight the possibility of developing a new generation of conjugated polymer/PET-based prosthetic devices that are highly biocompatible and potentially suitable for subretinal implantation in patients suffering from degenerative blindness.

## Introduction

The development of therapeutic approaches for retinal degenerative diseases is a hot spot in vision science research (Scholl et al., 2016). In fact, the progressive degeneration of retinal photoreceptors (Wright et al., 2010; Dias et al., 2018) is a public health issue with a concerning incidence worldwide (Hartong et al., 2006; Wong et al., 2014). Although one gene therapy for RPE65-linked retinal dystrophies is available (Russell et al., 2017), clinical interventions to treat other widespread retinal dystrophies based on optogenetics (Simunovic et al., 2019) and stem cells (Maeda et al., 2019) are still in preclinical experimentation and/or in early phases of testing in humans.

The wide variety of genetic defects causing retinal degeneration poses some challenges for the development of therapeutic approaches. Therefore, alternative treatments for these pathologies have been reported (Frasson et al., 1999; Busskamp et al., 2010; Leveillard and Sahel, 2010; Pearson et al., 2012; Barber et al., 2013). Among them are strategies that aim at rescuing vision by using electronic devices able to stimulate spared retinal networks (Walter et al., 2005; DeMarco et al., 2007; Gerding et al., 2007; Yanai et al., 2007; Zrenner et al., 2011; Mathieson et al., 2012; Mandel et al., 2013; Ayton et al., 2014; Lorach et al., 2015). However, strategies that use microelectrode-based stimulation (Humayun et al., 2012; Dagnelie et al., 2017) or photodiodes-driven chips (Stingl et al., 2013; Lorach et al., 2015) need power supply and external camera. Moreover, mechanical mismatch with retinal tissues, poor biocompatibility, high resistance and heat production represent further drawbacks. The optimization of the current retinal prostheses is therefore necessary to restore high-quality visual capabilities in dystrophic patients.

Conjugated polymers have been recently introduced as interfaces for neuronal stimulation (Ghezzi et al., 2011, 2013; Gautam et al., 2014; Martino et al., 2015; Feyen et al., 2016). A few years ago, we reported the ability of a planar, photovoltaic, biocompatible, and fully organic retinal prosthesis to persistently restore visual functions in a rat model of progressive neuronal degeneration, the Royal College of Surgeons (RCS) rat (Antognazza et al., 2016; Maya-Vetencourt et al., 2017). Not only sensitivity to light significantly increased, but also spatial resolution and metabolic activity of the visual cortex were positively affected. More recently, we developed a second-generation nanoscale prosthesis that similarly reinstates visual functions in dystrophic RCS animals (Benfenati and Lanzani, 2018; Maya-Vetencourt et al., 2020). In the present work, we directed our efforts toward the transfer of the initial results obtained in rodents to the swine model, as a preliminary step before phase-1 experimentation in humans.

As compared to rodents, we first enlarged the active surface of the prosthesis in consideration of the elevated retinal surface in pigs. Then, we analyzed various types of substrates for the photoactive polymers, which satisfied the needs of the surgical implant procedures. We first considered electro-spun silk fibroin (ESF) to have prostheses with a broader area, a high conformability and a more uniform surface, with respect to the ones previously used in RCS rats fabricated with crystallized silk fibroin (Maya-Vetencourt et al., 2017). Unexpectedly, the ESF caused a strong phlogistic reaction of the pig retina resulting in proliferative vitreoretinopathy (PVR). We therefore decided to use polyethylene terephthalate (PET) as substrate. Before the experimental tests in naive pigs, we confirmed that PET is an excellent biocompatible substrate in dystrophic rodents. The experimental results in pigs show the optimization both in shape and chemical composition of this novel retinal prosthesis and the set-up of a surgical implantation procedure ready to be translated to humans. Our findings demonstrate the biocompatibility and functionality of the device 10–30 days after implant in naive pigs.

## Materials and Methods

### Engineering of the Active Layers

The planar implants were created using the spin coating technique. The first conductive layer was obtained spin coating a PEDOT:PSS water dispersion (Clevios PH-1000) containing specific additives over the substrate. Briefly, the additives used were dimethysulfoxide (DMSO, 9% v/v) to increase the overall electrical conductivity, the surfactant Zonyl FS-300 (0.18% v/v) to promote dispersion wettability and facilitate the spin-coating process, the crosslinker 3-glycidoxypropyltrimethoxysilane (GOPS, 0.9% v/v) to enhance the adhesion of the PEDOT:PSS layer to the substrate and avoid delamination. The solution was deposited twice at the speed of 1600 rpm and treated for 300 s at 120°C. The top layer of the implants was created using poly(3-hexylthiophene) (P3HT), a polymer widely recognized in the literature and used for the manufacture of planar prosthetic devices of photovoltaic nature (Ghezzi et al., 2013; Gautam et al., 2014; Antognazza et al., 2016; Feyen et al., 2016; Maya-Vetencourt et al., 2017). The polymer was dissolved in chlorobenzene (30 mg/ml) and subsequently deposited at the speed of 1600 rpm. This way, the resulting films have a thickness of 200 nm and good homogeneity. The overall thickness of PEDOTS:PSS and P3HT layers obtained with this process is 550 nm.

### Engineering of the Prosthesis Substrate

We used two alternative substrates to support the polymeric layer, ESF and PET with distinct characteristics of flexibility, bending behavior. ESF, made from the same substrate used to fabricate the devices implanted in the rat (crystallized silk fibroin), provides an exceptionally uniform and flexible surface devoid of deformation memory.

Electro-spun silk fibroin substrates were produced as previously described (Alessandrino et al., 2008). Briefly, silk fibroin was autoclaved at 120°C, dissolved in LiBr (9.3 M) for 3 h at 60°C, and dialyzed against distilled water. The water solution has been drop-casted on petri dishes and the resulting films have been again dissolved in formic acid. The formic acid silk fibroin solution has been loaded in the electrospinning apparatus comprising two high voltage generators (F.u.G. Elektronik GmbH – HCN 35-12500), a syringe pump (Graseby Medical – MS 2000), and a stainless steel 0.5 mm wide spinneret. Positive potential was applied to spinneret and negative potential to the collector plate. After the deposition, the electro-spun films were detached from the collector plate treating with water at 37°C for 5–10 min and isopropanol for 10 min, dried, and then treated with methanol to induce silk fibroin crystallization. The resulting fibroin fabric was characterized by an excellent layering of polymers and an easy curling for introduction in the posterior chamber of the pig eye, with the possibility to significantly increase of coverage area of the retina with respect to more rigid substrates.

Polyethylene terephthalate substrates, whose biocompatibility has already been widely verified in the biomedical field, were obtained from 23 μm thick films (Hostaphan^®^, Mitusbishi Polyester Film). Before use, the material was carefully cleaned through three sonication baths using milli-Q water, acetone and isopropanol. Substrate films were subjected to plasma treatment in air at 40 W to improve the adhesion of the polymeric layers.

We used laser cutting to give the devices their final shape. The light source was an amplified mode-locked Yb:KGW laser with emission at 1030 nm. The laser was frequency-doubled using a HIRO harmonic generator and operated at 500 KHz with a pulse width of 240 fs. The light beam was focused using a 20x objective and the incident power was 100 mW. Samples were mounted between two glass slides and placed on a three-axis micro-controlled stage; the stage was moved at 0.4 mm/s. The cut was repeated 10 times at different z-positions to facilitate the detachment of the sample from the glass slides. The final cut width was below 10 μm.

### Experimental Animals and Ethical Considerations

The use of domestic pigs in this study (total *n* = 8 of either sex; approx. 3 months of age) was regulated by two protocols approved by the Italian Ministry of Health (D.Lgs 116/92 and D.Lgs 26/2014) in accordance with the Association for Research in Visual Ophthalmology Statement for the Use of Animals in Ophthalmic and Vision Research. Commercial hybrid pigs [(Large White × Landrance) × Duroc] were enrolled in the study and housed in multiple boxes according to their origin dominance group to reduce stress and aggressiveness. Animals, kept at 20 ± 4°C with a 12/12 h light/dark cycle were fed with standard swine diet (CESAC s.c.a. Conselice RA, Italy) twice a day and received water *ad libitum* (Barone et al., 2020). Chains and pieces of wood were used as environmental enrichment; no bedding materials were dispensed to guarantee good health conditions; every 2 days the animals received a small amount of straw as rooting material (Barone et al., 2018). Royal College of Surgeons inbred, pink-eyed dystrophic (RCS) rats (total *n* = 51; either sex; approx. 3 months of age) and congenic non-dystrophic controls (RCS-rdy) were provided by M. M. La Vail (Beckman Vision Center, University of California, San Francisco, California) (LaVail and Battelle, 1975). All manipulations and procedures were performed in accordance with the guidelines established by the European Community Council (Directive 2012/63/EU of 22 September 2010) and were approved by the Italian Ministry of Health (Authorization no. 645/2015-PR). RCS rat colonies were bred under standard conditions with food and water *ad libitum* under a 12:12 h light/dark cycle, as previously described (Maya-Vetencourt et al., 2017).

### Surgical Procedures in the Pig

Domestic pigs were weighted and sedated with an intramuscular (IM) injection of Tiletamine-Zolazepam (Zoletil, Virbac, Prague, Czechia) (5 mg/kg). After 15 min, general anesthesia was induced with 8% sevoflurane (SevoFlo, Esteve, Barcelona, Spain), administered through a mask in a 1:1 oxygen/air mixture and maintained with the same halogenated agent (3%) after orotracheal intubation (Elmi et al., 2019). Lactated Ringer infusion was set at a rate of 10 ml/kg/h through the auricular vein (left ear). Heart frequency rate, non-invasive blood pressure (NIBP), peripheral capillary oxygen saturation SpO_2_ and CO_2_ were strictly monitored along anesthesia (Ventrella et al., 2017). Local analgesia was granted by the application of oxybuprocaine hydrochloride eye drops (Novesina 0.4%, Visufarma s.r.l. Rome, Italy).

After shaving and disinfecting the periocular area with Povidone-Iodine 10%, a sterile drape was positioned and the eye exposed with a speculum. Two 23-gauge and one 20-gauge trocars were inserted 1.5 mm from the limbus and a chandelier light was positioned to allow bimanual surgery. After the induction of a posterior vitreous detachment by active suction of the cutter, vitrectomy was performed, paying attention to avoid the lens. The posterior retina was separated from the retinal pigmented epithelium (RPE) by injecting a balanced salt solution (Balanced Salt Solution; Alcon Laboratories, TX, United States) into the subretinal space through a 41-gauge subretinal cannula connected with the automated pump of the machine. One injection was fine to detach the temporal side of the visual streak. A 1.5 mm mid-peripheral retinotomy was performed with scissors (DORC International). The implant was inserted in the vitreous cavity through an injector, specifically designed for this purpose, that fitted on the 20-gauge trocar. Subsequently, it was inserted into the subretinal space using two intraocular forceps. Perfluorocarbon liquid (PFCL) was injected into the vitreous cavity enough to cover the retinotomy, drain the subretinal fluid and reattach the retina. Laser endophotocoagulation was performed around the edge of the retinotomy. A fluid/air exchange was carried out. The trocars were removed and the sclerotomies sutured with Vicyl 8-0. An isovolemic mixture of Sulfur Hexafluoride (SF6) gas was used as final tamponade. After surgery, all animals received an intra-conjunctival administration of triamcinolone acetonide (40 mg per eye, Kenacort, Bristol-Myers Squibb, New York, United States) and fuocinolone acetonide/neomycin eye drops (Iridex, Ceva Vete, Agrate Brianza, Italy).

### Surgical Procedures in the RCS Rat

Three months-old RCS rats of either sex were anesthetized with an intraperitoneal injection of diazepam (10 mg/kg) followed by intramuscular administration of xylazine (5 mg/kg) and ketamine (33 mg/kg). The sub-retinal implant of the prosthesis was carried out in a sterile room using a Leica ophthalmic surgical microscope, as previously described (Maya-Vetencourt et al., 2017). Briefly, a 1.5 mm peritomy was made 2 mm posterior to the limbus, followed by a limbus-parallel incision through the sclera and choroid of approximately 1 mm. The retina was then gently detached from the choroid using a custom-designed spatula. Viscoelastic material was injected into the subretinal space and the device was inserted with the polymeric coating facing the retina. The edges of the sclerotomy were electrocoagulated and the conjunctiva apposed. At the end of the surgical intervention, the position of the device was evaluated by indirect ophthalmoscopy and antibiotic and cortisone were applied to both eyes.

### Optical Coherence Tomography Analysis

Pigs were anesthetized as described above and their pupils dilated with 1% tropicamide eye drops (Visumidriatic, Visufarma s.p.a. Rome, Italy) before image acquisition. Spectral domain optical coherence tomography (*SD*-OCT) was performed using the iVue device (Optovue, Fremont, United States) using 840 ± 10 nm light with power at the pupil of 750 μW. The depth of resolution in tissue is 5 μm, and the transverse resolution is 15 μm. Each image covered a 6 × 6 mm area centered on the fovea, acquired at 26.000 A-scan/second and composed of 256 to 1024 A-scan/frame.

### Electrophysiological Recordings

The electrophysiological analysis in pigs was performed before and after the implant procedure, with the animals under general anesthesia induced with the protocol described above. Pupils were dilated with 1% tropicamide eye drops (Visumidriatic, Visufarma s.p.a. Rome, Italy). Two drops of oxybuprocaine hydrochloride (Novesina, Visufarma s.r.l. Rome, Italy) were used as a local anesthetic, then a barraquer blepharostat was used and eyes were maintained in a central and stable position by two stay sutures. Corneal contact lenses electrodes (ERGjet, Universo Plastique, Switzerland) were used as active electrodes while dermal needle electrodes were used as reference and ground, during ff-ERG. A drop of 3% carbomer (Dacriogel, Alcon, Fort Worth, TX, United States) was applied to the inner surface of the lens electrodes to protect the cornea and to ensure good electrical contact. The International Society for Clinical Electrophysiology of Vision (ISCEV) Standards for ff-ERGs were used with the settings adjusted for the swine species, as previously described (Barone et al., 2020). We performed ff-ERG recordings using the light-adapted ERG 3.0 protocol because the retinal implant was surgically positioned in high cone density visual streak of the swine retina (Hendrickson and Hicks, 2002). Briefly, ERG recordings were filtered with a 2–500 Hz band-pass FFT filter. For ff-ERGs, the amplitude (μV) from “a” to “b” waves was measured. The adaptation/background light luminance and time were: 30 cd/m^2^ and 10 min. The ff-ERG stimuli were produced by a Ganzfield dome and the patterned stimuli by a screen connected to a pattern generator. The stimulus luminance was of 3 cd/s/m^2^ with an inter-stimulus time of 1.1 Hz. The data were amplified and acquired by the WinAverager Software. The Ganzfield dome, the pattern screen and generator, the amplifier, and the Software used were part of the BM6000-MAXI Electrophysiology Unit (Biomedica Mangoni, Pisa, Italy). Both eyes were separately investigated while covering the opposite one to prevent light exposure during the electrophysiological recordings. Each experimental session, including the animals’ preparation, lasted about 2 h. At the end of the electrophysiology sessions, the animals were gently recovered from anesthesia. The electrodes, the sutures and the blepharostat were removed and Chloramphenicol 10 mg-Sodium colistimethate 180,000 U.I.-Tetracycline 5 mg ophthalmic ointment (Colbiocin; SiFi, Catania, Italy) was applied on the conjunctiva. The animals were then moved to a dark and quiet room and the orotracheal tube was removed when the swallowing reflex reappeared. Pigs completely recovered from anesthesia within a maximum of 2 h were then moved back to their origin pen. As to the RCS dystrophic rats, the electrophysiological recordings of visually evoked potentials (VEPs) were performed as reported in Maya-Vetencourt et al. (2017). In the case of light sensitivity, visual stimuli were flashes of light (20 cd.m^–2^; 100 ms) whereas in the case of spatial acuity, visual stimuli were horizontal sinusoidal gratings of increasing spatial frequencies (0.1–1) cycles per degree of visual angle (c.deg^–1^) at 0.5 Hz. Light sensitivity corresponded to the amplitude of the VEP response to flashes of light. Spatial acuity was obtained by extrapolation to zero (0) amplitude of the linear regression through the last 4–5 data points of VEP amplitude against the log spatial frequency. Electrophysiological traces were reconstructed and analyzed using the OriginPro 9.1 software.

### Immunohistochemistry

At the end of the experimental protocol, pigs were euthanized and eyes were immediately explanted and fixed with 4% paraformaldehyde buffer; a small incision at the limbus level was made to improve fixative permeability. After cornea and lens removal, each eye was cut in four quadrants (superior, inferior, temporal and nasal), embedded in mounting medium (Tissue Tek OCT compound; Sakura Finetek, Torrance, CA, United States) and snap-frozen in liquid nitrogen. Cryosections were cut at 20 μm (CM1850 Cryostat; Leica, Wetzlar, Germany) and collected on gelatin and poly-L-lysine coated slides. Retinal sections were incubated with 10% bovine serum albumin (BSA, Merk, Darmstadt, Germany) to block non-specific binding sites and immunolabeled overnight at 4°C with primary antibodies (mouse monoclonal anti-FGF2 antibody, 1:200, Merk, Darmstadt, Germany; rabbit polyclonal anti-GFAP, 1:5000, Dakocytomation, Campbellfield, Australia; rabbit anti-IBA-1, 1:1000, Wako, Richmond, United States; mouse anti-Rhodopsin 1:200, Thermofisher, Waltham, United States). Tissues were then incubated with appropriate secondary antibodies conjugated to a fluorescent dye (Alexa Fluor 488, Alexa Fluor 594; Molecular Probes, Invitrogen, Carlsbad, CA, United States) for 2 h at 37°C. Nucleus staining dye, 4′,6-diamidino-2-phenylindole (DAPI, Merk, Darmstadt, Germany), was used before slides coverslips apposition. Images were acquired with confocal microscopy (Leica TCS SP5 II) and analyzed with Fiji software (Wayne Rasband, NIH, United States). Photoreceptors’ survival was estimated by measuring the outer nuclear layer (ONL) thickness in DAPI-stained images. Quantification of fluorescence was obtained by measuring the integrated fluorescence intensity acquired from equivalent ROIs for FGF2 and IBA-1 in the entire ONL and for GFAP throughout the retina. The number of IBA-1-positive cells was manually evaluated by counting the nuclei both in the ONL and in the entire retina. The morphology of IBA-1-positive microglial cells was evaluated via Scholl analysis of sampled cells from the entire retina by using the free software Fiji default plugin.

### Statistical Analysis

The statistical analysis was performed using the Prism 6.0 software (GraphPad Prism, San Diego, CA, United States). The experiments of ff-ERG responses (PRE and POST) in pigs implanted with the PET-based device were analyzed using the paired Student’s *t*-test. The analysis of visual functions (light sensitivity and visual acuity, respectively) in dystrophic RCS rats implanted with the PET-based device (single variable experiments) was performed using the one-way ANOVA/Tukey’s test. The histological morphology of the retina, the astrocytic and microglial responses as well as the FGF2 expression in the pig retina after implantation of the PET-based device were analyzed, in different retinal areas (two variable experiments), using the two-way ANOVA/Tukey’s test. Differences with *p* < 0.05 were considered significant. The results were expressed as means ± SD of the indicated *n* values. Regarding non-implanted naïve pigs and animals implanted with the ESF-based prosthesis, the data include an *n* = 2 in each experimental design. As to the electrophysiological, histological, and immunohistochemical analysis, the data of pigs implanted with the PET-based prosthesis correspond to 4 independent animals each implanted in the left eye with the active device and in the right eye with the sham material. The data of controls include 4 eyes obtained from 2 naïve pigs.

## Results

### Prosthesis Engineering and Surgical Implant and Recording Procedures in the Pig

**FIGURE 1:**
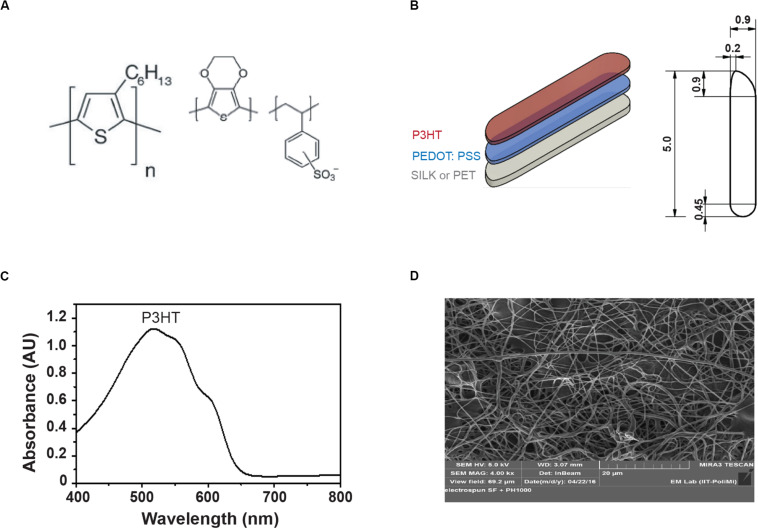
Characterization of the polymeric prosthesis. **(A)** Structural formulas of the two polymeric components of the prosthetic device, the semiconductive P3HT (left) and the organic conductor PEDOT:PSS (right). **(B)** The figure depicts the layer composition and the dimensions (mm) of the prosthesis designed for the pig’s eye. **(C)** Absorbance spectrum of the P3HT layer in the prosthesis shows its activation profile extending to the majority of the visible spectrum. **(D)** SEM image of the filamentous ultrastructure of the ESF substrate (magnification, 4000x; view field, 69.2 μm).

Conductive (PEDOT:PSS) and semiconductive (P3HT) polymers (Figure 1A) were successively layered on top of the substrate of choice as described in the methods and the complete device was subjected to laser-assisted cutting with rounded edges to the final dimensions shown in Figure 1B. The prostheses in the final configuration showed an absorption spectrum that covered the entire visible spectrum with a peak in the green region (Figure 1C). ESF membrane used as passive substrates display at the scanning electron microscopy analysis a mesh of intermingled thin fibroin fibers (Figure 1D).

The optimization of the surgical procedure for the implant of the retinal prosthesis then took place. Since the first interventions conducted in pigs using the same procedure previously followed in rats (*trans*-scleral implantation) presented problems in the post-operative phase, we decided to access the retina through the posterior chamber. Thus, to introduce a sufficiently large device, the prosthesis substrate should be rolled up for insertion of the implant into the 20-gauge trocar, followed by unrolling inside the eye’s posterior chamber.

**FIGURE 2:**
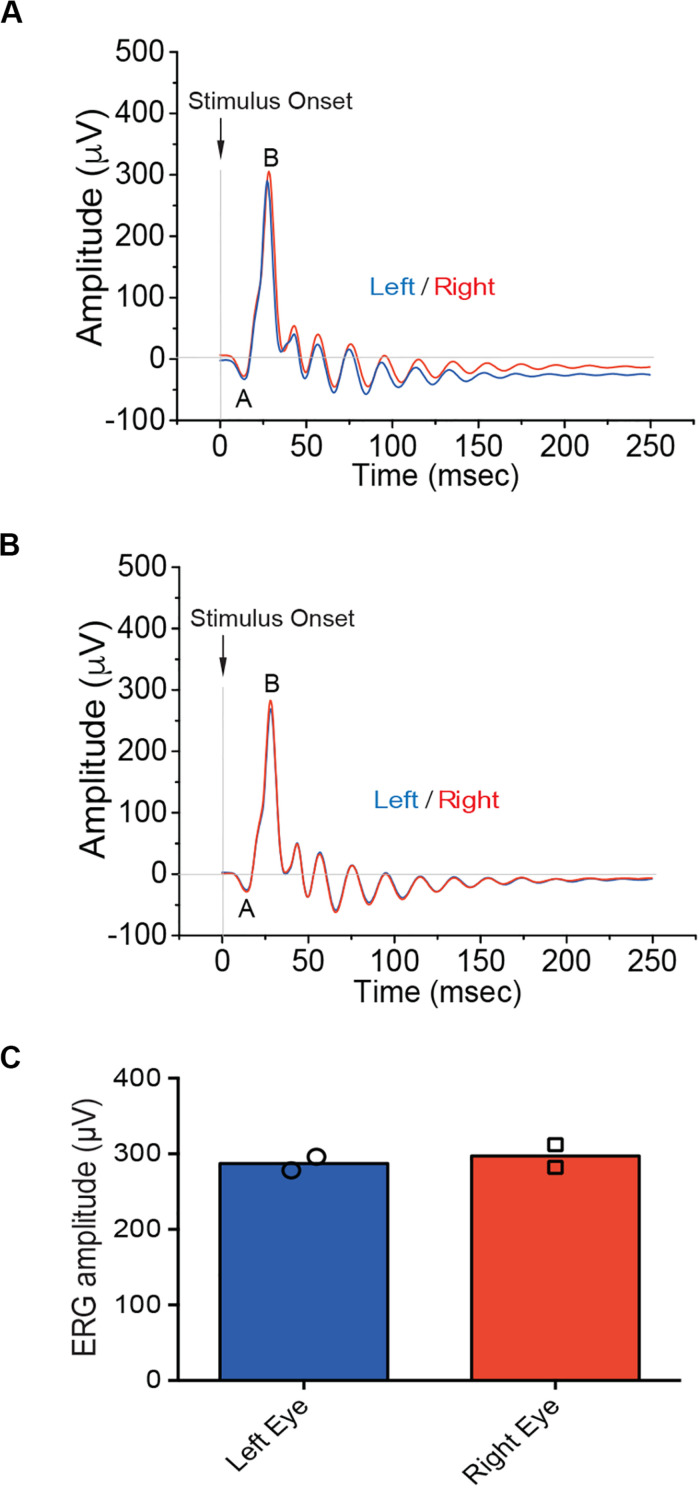
Electroretinographic responses in naïve pigs. **(A,B)** Representative ff-ERG recordings in the left (blue trace) and right (red trace) eyes, respectively, from the G1 **(A)** and G2 **(B)** naïve pigs (*n* = 2). **(C)** Bar plots (means with superimposed experimental points) depicting the amplitude of the ff-ERGs b-wave (μV) in the left and right eyes of the G1 and G2 pigs. The electrophysiological recordings of ff-ERGs show that b-wave amplitudes are not different in both naïve pigs validating the experimental approach used to assess retinal functionality.

To setup a standard evaluation of retina activation and physiological integrity, electroretinography was used in naïve pigs. Clear ff-ERG traces were obtained in adult anesthetized naïve animals. As expected, ff-ERG b-wave signals corresponding to the left and right eyes in the same animal were closely similar in both amplitude and latency (Figures 2A,B) and similar traces were obtained from independent experimental pigs (Figure 2C). This was confirmed by the *SD*-OCT images obtained immediately after the electrophysiological analysis, which showed full integrity of retinal layers (Supplementary Figure S1A).

### Implant of the Polymeric Device on ESF in the Pig

Since crystallized silk fibroin was demonstrated to be a harmless passive substrate after implantation in RCS rats (Antognazza et al., 2016; Maya-Vetencourt et al., 2017), we initially thought to adopt the same material for manufacturing the larger prostheses to implant in the pig’s eye. However, crystallization could not achieve large planar surfaces. In addition, large crystallized fibroin surfaces do not have sufficient compliance to adapt to the retina curvature. For this reason, keeping the same source material, we generated electro-spun fibroin fibers organized in a mesh (Figure 1D) that was similar in behavior to silk fabrics. The resulting fibroin fabric was characterized by an excellent layering of polymers, a facilitated folding and unfolding during the surgical implant, with high potential to cover large areas of the retina.

**FIGURE 3:**
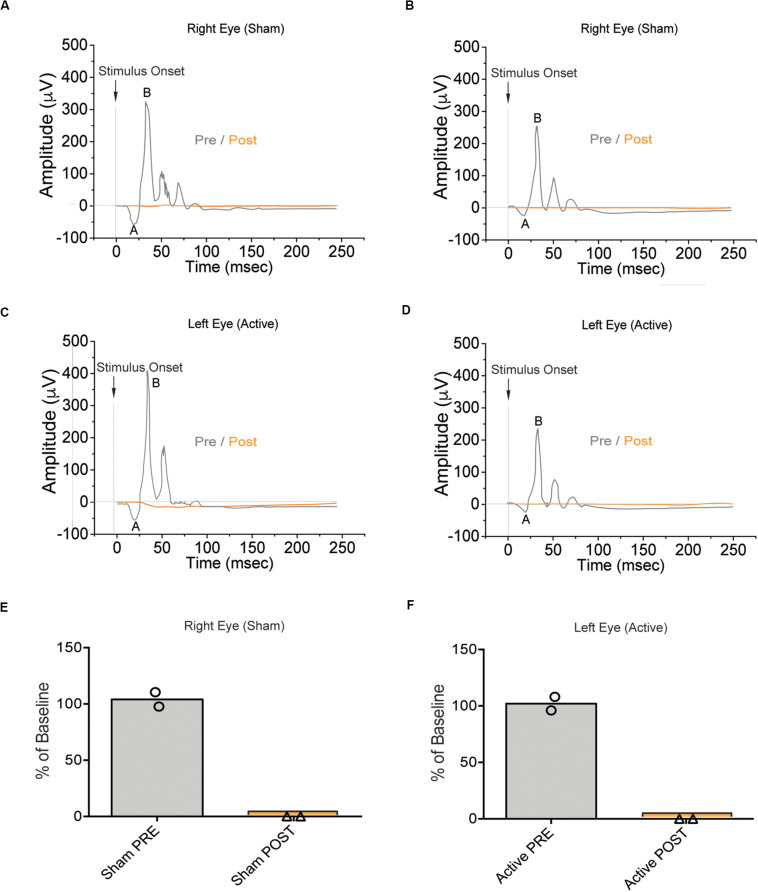
Electroretinographic responses in naïve pigs subretinally implanted with the ESF-based substrate. **(A,B)** Representative ff-ERG signals of the right eye of the E2 (left) and E3 (right) pigs before surgery (Pre, gray) and after (Post, orange) implantation of the sham ESF substrate alone. **(C,D)** Representative ff-ERG signals of the left eye of the E2 (left) and E3 (right) pigs before surgery (Pre, gray) and after (Post, orange) implantation of the ESF-based complete polymeric device (active). **(E,F)** Bar plots (means with superimposed experimental points) depicting the amplitude of the ff-ERGs b-wave before (Pre, gray) and after (Post, orange) the implantation of either sham substrate (right eye; E) or the active device (left eye; **F**) in the E2 and E3 pigs (*n* = 2). Data are expressed as the percentage of baseline. The use of ESF as a substrate, irrespective of whether it was layered with the polymers or bare-implanted, had a detrimental impact on the visual response to light after implantation.

**FIGURE 4:**
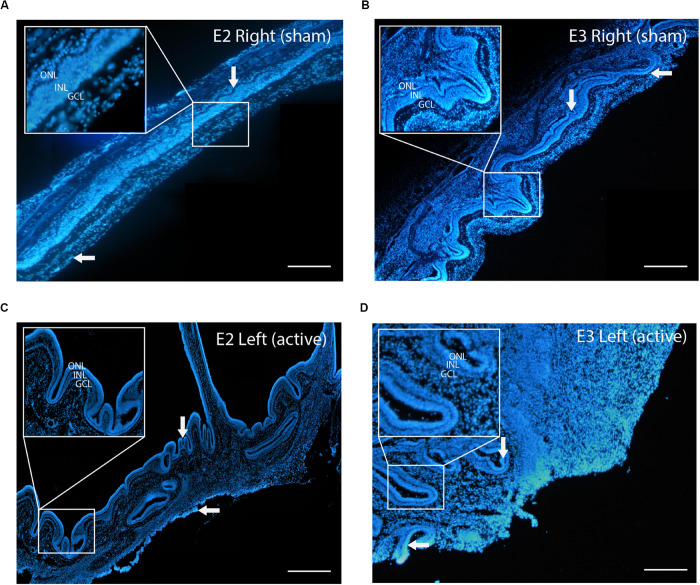
Proliferative vitreoretinopathy (PVR) to the implanted ESF substrate. **(A,B)** Bisbenzimide staining of the retina dissected from the right eyes of the E2 (left) and E3 (right) pigs that were sham-implanted with the ESF substrate (*n* = 2). **(C,D)** Bisbenzimide staining of the retina dissected from the left eyes of the E2 (left) and E3 (right) pigs that were implanted with the ESF-based polymeric device. *Upper insets:* structure of the retinal layers at higher magnification. In all images, a marked PVR that includes the formation fibrin and curling of the retina is present, witnessing an intense inflammatory reaction to ESF-containing devices. Ocular media opacities were also found by indirect ophthalmoscopy. These phenomena correlate with the complete loss of the ff-ERG signals (see Figure 3). *Downward arrow*: curling of the retina. *Leftward arrow*: formation of fibrin. Scale bars, 150 μm.

Although this material had ideal physical-chemical characteristics to adopt an endo-vitreal approach, the experimental findings revealed that it was poorly biocompatible with the pig’s retina and triggered adverse reactions. Indeed, the electrophysiological analysis following the surgical implant of 4 aligned ESF-based prosthesis per eye, showed a complete absence of the ff-ERG b-wave signal after 10 days of implant, from both the eyes implanted with the complete device (active; left eye) and the eyes implanted with the substrate only (sham; right eye) in the two tested animals (E2 and E3 pigs; Figures 3A–D), as compared to the respective initial recording sessions (PRE vs. POST; Figures 3E,F). Indeed, in the days following the operation indirect ophthalmoscopy and *SD*-OCT scans revealed marked alterations in outer retinal layers in direct contact with the prosthesis (Supplementary Figure S1B). Retrospective histochemistry confirmed the existence of an intense PVR with proliferation, curling, and incorporation of the device inside the reactive tissue (Figures 4A–D), in contrast with the excellent biocompatibility previously demonstrated for crystallized fibroin in the rat (Antognazza et al., 2016). Since the retinal reaction occurred in both eyes implanted with active or sham devices, it is possible that the local inflammatory reaction was caused by either the filamentous microstructure of ESF or the progressive fibroin swelling and subsequent curling of the device.

### Replacement of ESF With PET and Functional Testing in the RCS Rats

**FIGURE 5:**
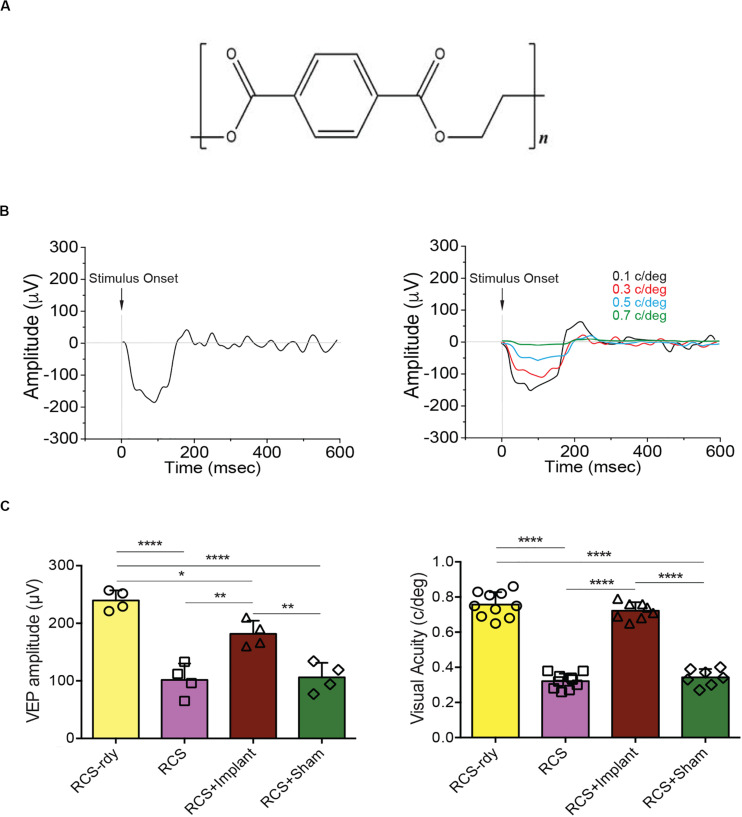
Light sensitivity and visual acuity in adult dystrophic RCS rats implanted with the PET-based polymeric prosthesis. **(A)** Structural formula of polyethylene terephthalate (PET). **(B)** *Left:* representative trace of the visual evoked potential (VEP) recorded in the binocular area of the primary visual cortex in non-dystrophic RCS-rdy rats. *Right:* representative VEP traces in non-dystrophic RCS-rdy rats showing the progressive decrease of the signal amplitude that occurs with the increase of the spatial frequency of the visual stimulus. **(C)** *Left:* the VEP response to light in naïve RCS dystrophic rats (102 μV) was lower (*p* < 0.0001) than that observed in RCS-rdy congenic controls (240 μV). Sensitivity to light increased significantly (182 μV) after 1 month of implant with the active device as compared to either non-implanted dystrophic RCS rats (*p* = 0.0024) or sham-implanted dystrophic RCS rats (*p* = 0.0038). Light sensitivity in sham-implanted RCS animals did not differ from that seen in naïve RCS rats (*n* = 4 per experimental group). *Right:* visual acuity in naïve dystrophic rats (0.32 c/deg) was lower (*p* < 0.0001) than that of RCS congenic controls (0.76 c/deg) but following 1 month of implantation with the PET-based prosthetic it significantly enhanced (0.72 c/deg) with respect to either naïve RCS animals (*p* < 0.0001) or sham-implanted RCS rats (*p* < 0.0001). Data are means ± SD with superimposed experimental points. *p < 0.05; **p < 0.01; ****p < 0.0001; one-way ANOVA/Tukey’s tests (*n* = 10 for both RCS-rdy and RCS animals; *n* = 8 for RCS + Implant; *n* = 7 for RCS + sham).

In light of these findings, we decided to modify the nature of the passive substrate by using PET (Figure 5A). Although the biocompatibility of this material is well known, and PET is widely used in biomedical applications, we conducted preliminary functional tests in RCS dystrophic rats implanted for 1 month with a prosthetic device with the previously described geometry specifically designed for the rat eye (Maya-Vetencourt et al., 2017). To this end, electrophysiological recordings of visual evoked potentials (VEPs) in the binocular area of the primary visual cortex of dystrophic RCS animals were performed, as previously described (Maya-Vetencourt et al., 2017) by implanting either the complete device, or PET alone (sham). The amplitude of VEP responses to light flashes or the spatial discrimination of the responses evoked by patterned stimuli in implanted animals were compared with non- or sham-implanted dystrophic RSC rats and congenic non-dystrophic RCS-rdy rats of the same age (Figure 5B). This analysis revealed the capability of PET-based retinal prostheses to improve visual functions in rats with neurodegenerative retinal dystrophy. Indeed, a significant enhancement of both light-sensitivity and spatial acuity (Figure 5C) was observed in RCS implanted rats as compared to both naïve and sham-implanted RCS controls, respectively. The functional rescue was in the same order of magnitude of that obtained using crystallized silk fibroin as substrate (Maya-Vetencourt et al., 2017), indicating that the passive substrate does not directly affect the activity of the overlying polymeric layers.

### Implant of the PET-Based Polymeric Device in Pigs

**FIGURE 6:**
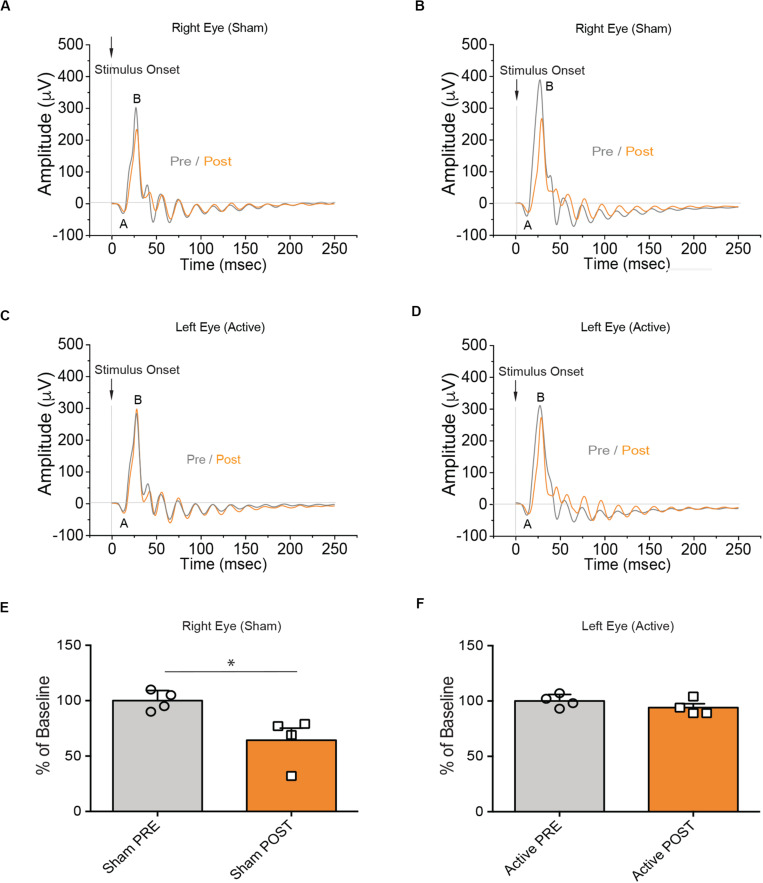
Electroretinographic responses in naïve pigs subretinally implanted with the PET-based prosthesis. **(A,B)** Representative ff-ERG signals of the right eye of the E31 (left) and E34 (right) pigs before surgery (Pre, gray) and after (Post, orange) implantation of the sham PET substrate alone. **(C,D)** Representative ff-ERG signals of the left eye of the E31 (left) and E34 (right) pigs before surgery (Pre, gray) and after (Post, orange) implantation of the PET-based complete polymeric device (active). **(E,F)** Bar plots (mean ± SD) depicting the amplitude of the ff-ERGs b-wave before (Pre, gray) and after (Post, orange) the implantation of either sham substrate (right eye; **E**) or the active device (left eye; **F**) in *n* = 4 individual pigs. Data (means ± SD with superimposed experimental points) are expressed as the percentage of baseline. Before the surgical intervention, the amplitude of the ff-ERG b-wave in the eyes of all pigs was normal. Notably, these signals were preserved after the subretinal implantation of the PET-based active prosthetics in all animals (*p* = 0.1492). No rescue effect was observed in sham-implanted animals (*p* = 0.0182). ^∗^*p* < 0.05, paired Student’s *t*-test (*n* = 4).

After optimizing the chemical composition of the prosthesis and testing it in rodents, we implanted 4 aligned pieces of PET-based devices per eye to increase retina coverage in the swine model. Unlike the ESF-based prosthesis, PET-based devices did not elicit overt adverse reactions in the retina of naive pigs (Figure 6). In the four analyzed pigs, the amplitude of the ff-ERG b-wave was slightly, but significantly, decreased (*p* = 0.0182, paired Student’s *t*-test) after the surgical procedure in the sham-implanted eye (Figures 6A,B,E). Notably, in the four eyes implanted with the active prosthesis, ff-ERG b-wave amplitudes did not significantly differ before and after the surgical procedure (*p* = 0.1492, paired Student’s *t*-test) (Figures 6C,D,F). No significant difference in the latency of the ff-ERG b-wave signal was observed. In addition, *SD*-OCT scans obtained after the electrophysiological analysis, revealed full integrity of the retinal layers that were in tight contact with the implant (Supplementary Figure S1C,D). The decreased post-implant ERG responses in sham-implanted eyes, are consistent with a partial surgery-evoked impairment. On the other hand, the preservation of the responses in eyes implanted with active devices, which underwent the same surgical implantation procedure, may signal an active compensation by the implanted device (Figures 6E,F).

### Biocompatibility of the PET-Based Implant

**FIGURE 7:**
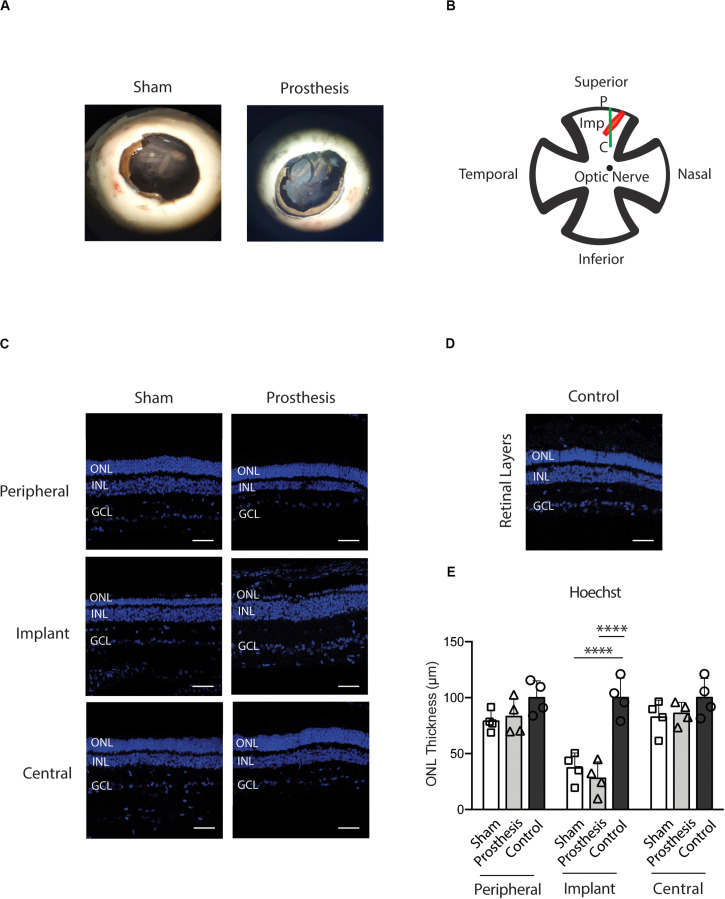
Morphology of the ONL after implantation of PET-based devices in the pig. **(A)** Representative eyecups of sham- and prosthesis-implanted pigs. All the examined eyes had no overt signs of inflammatory reaction or displacement of the implants from the insertion place. **(B)** Cartoon depicting the retinal areas (“peripheral,” “implant,” and “central”) that were sampled in sham- and prosthesis-implanted eyes. **(C,D)** Representative retina sections stained with bisbenzimide to show the thickness of nuclear layers in “peripheral,” “implant,” and “central” positions in sham- and prosthesis-implanted pigs **(C)** as well as in the non-implanted control group **(D)**. ONL, outer nuclear layer; INL, inner nuclear layer; GCL, ganglion cell layer; P, Peripheral; Imp, implant; C, central. Scale bars, 50 μm. **(E)** Bar plots (means ± SD with superimposed experimental points) showing the retinal ONL thickness measured in sham-implanted (white), prosthesis-implanted (light gray) and non-implanted control (dark gray) groups measured in the peripheral, implant and central retinal regions. In the implant area, a significant reduction in the ONL thickness of the sham (*p* < 0.0001) and prosthesis (*p* < 0.0001) group with respect to non-implanted controls was observed. No significant changes in retinal thickness in the other retinal regions were found. ^****^p < 0.0001, two-way ANOVA/Tukey’s tests (*n* = 4 for each experimental group).

Based on the positive electrophysiological and OCT data, we next assessed in more detail the biocompatibility of the implanted PET-based device by analyzing the retina cellularity and performing immunohistochemical assays for various inflammatory/trophic markers. We compared untreated eyes collected from normal pigs with the eyes of animals that were subjected to bilateral surgery. After carefully dissecting the eyes, removing cornea, lens, and vitreous body, we checked the position of the implant and signs of a possible glial reaction caused by either the surgery or the chemical composition of the device. At the macroscopic level, we did not find marked signals of gliosis or signs of retinal detachment (Figures 7A,B). Both PET-based and sham devices were in subretinal position with no indication of movement after the insertion. To check for morphological changes or remodeling of the ONL due to the foreign body insertion between the photoreceptor layer and the RPE, we performed a nuclear staining with bisbenzimide in implanted (Figure 7C) and non-implanted (Figure 7D) eyes. We measured the ONL thickness in three positions of the retina: the area of the implant and the two neighboring areas of the Periphery/Optic disk axe that we refer to as “Peripheral” and “Central” areas (Figure 7B). An expected, thinning of the ONL in the “implant” area was observed in both active device and sham-device eyes, with no significant difference between the two eyes (Figure 7E). The thickness of the other two neighboring areas of the retina, in both sham-implanted and prosthesis-implanted eyes, was comparable to those of non-implanted animals (control; Figure 7E).

We then performed a series of immunohistochemical assays in sham- and prosthesis-implanted pig eyes to check for the expression of neuroinflammatory/trophic markers, namely: (i) the glial fibrillary acidic protein (GFAP); (ii) the ionized calcium-binding adapter molecule 1 (IBA-1); (iii) fibroblast growth factor 2 (FGF2) in the previously described “peripheral,” “implant” and “central” areas.

**FIGURE 8:**
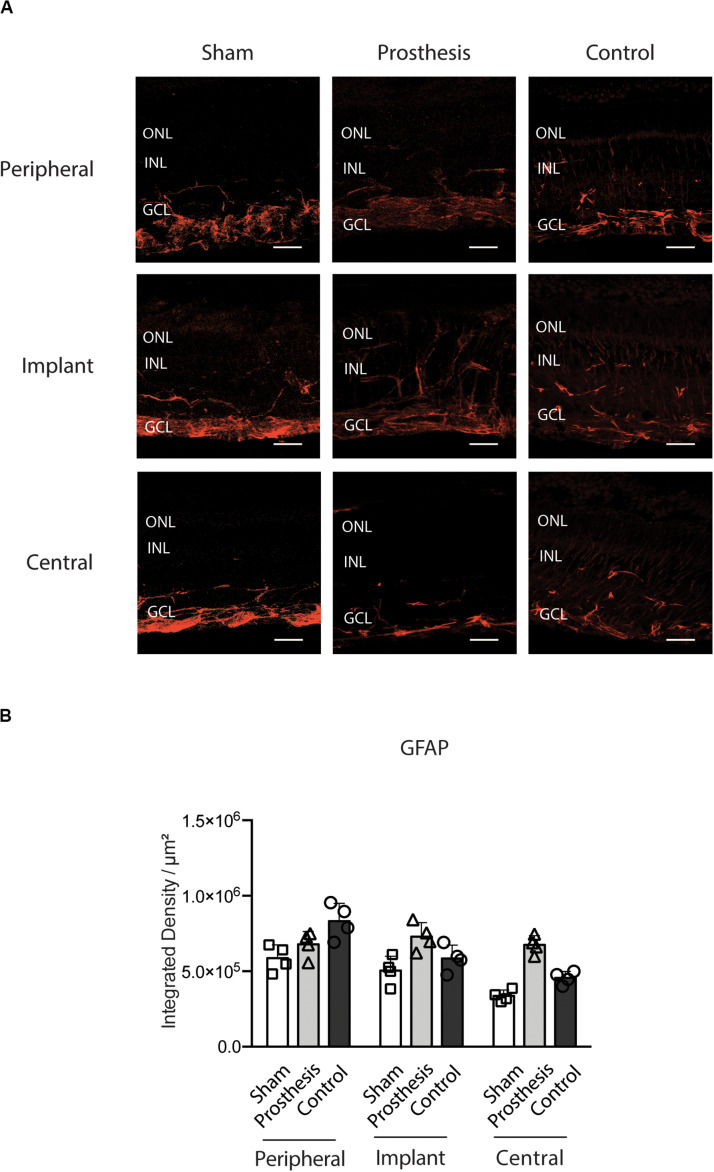
Astrocytic response in the retina after implantation of PET-based devices in the pig. **(A)** Representative retinal sections stained with anti-GFAP antibody (red), labeling astrocytes. The immunoreactive areas were sampled as indicated in Figure 7B. ONL, outer nuclear layer; INL, inner nuclear layer; GCL, ganglion cell layer. Scale bars, 50 μm. **(B)** Bar plots (mean ± SD) displaying GFAP levels in the retinas of sham-implanted (white), prosthesis-implanted (light gray) and non-implanted control (dark gray) groups measured in the peripheral, implant and central retinal regions. All retinal areas showed comparable GFAP levels (*p* > 0.05 for all comparisons between experimental groups; two-way ANOVA/Tukey’s test, *n* = 4), indicating the absence of a significant glial activation following the surgical implant.

Glial fibrillary acidic protein expression correlates with neuroinflammation (Brahmachari et al., 2006; Kim et al., 2016; Zhang et al., 2017). In the retina, GFAP is constitutively expressed in astrocytes and Muller cell’s soma; it is upregulated following stress and, in Muller cells, translocated along their outer and inner branches (Lewis and Fisher, 2003; Edwards et al., 2016; Luna et al., 2016; Zhang et al., 2017; Lu et al., 2018) (Figure 8A). No significant changes in GFAP levels were found between sham- or prosthesis-implanted and control animals (Figure 8B).

**FIGURE 9:**
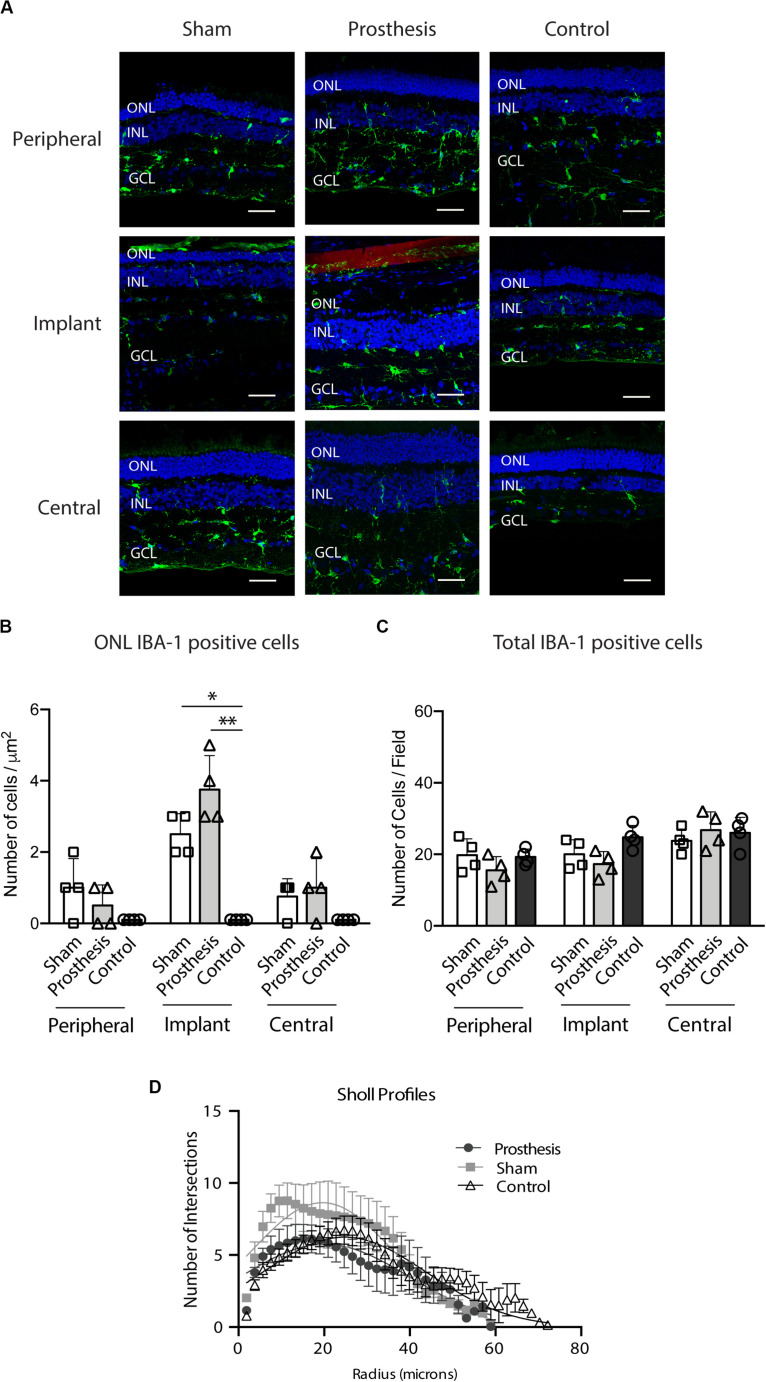
Microglial response in the retina after implantation of PET-based devices in the pig. **(A)** Representative retinal sections stained with anti-IBA-1 antibody (green) and bisbenzimide (nuclei, blue). The PET-based active device was detected thanks to the P3HT fluorescence (red). The immunoreactive areas were sampled as indicated in Figure 7B. ONL, outer nuclear layer; INL, inner nuclear layer; GCL, ganglion cell layer. Scale bars, 50 μm. **(B)** Bar plots (means ± SD with superimposed experimental points) depicting the number of IBA-1-positive microglial cells in the ONL of sham-implanted (white), prosthesis-implanted (light gray) and non-implanted control (dark gray) groups measured in the peripheral, implant and central retinal regions. In the ONL, all groups show a similar number of IBA-1-positive cells, except for a significant increase of microglia in the implant area of both sham- and prosthesis-implanted groups with respect to control retinas (*p* = 0.0266 and *p* = 0.0039, respectively). **(C)** Bar plots (means ± SD with superimposed experimental points) depicting the number of IBA-1-positive microglial cells in the entire retina of sham-implanted (white), prosthesis-implanted (light gray) and non-implanted control (dark gray) groups measured in the peripheral, implant and central retinal regions. The total number of cells is not significantly changed. **(D)** Scholl analysis of microglial processes shows the number of intersections of the processes of IBA-1-positive cells in concentric circles centered on the cell body and drawn every 0.38 μm. Morphological shapes are similar in all experimental groups, indicating the absence of a significant microglia activation. ^∗^*p* < 0.05; ^∗∗^*p* < 0.01; two-way ANOVA/Tukey’s tests (*n* = 4).

Microglia are a resident glial cells population. They represent tissue macrophages that, in healthy retinas, play an essential role in homeostasis. Their normal localization is in the plexiform layers, GCL and nerve fiber layer, where they are responsible for immune surveillance and synapses homeostasis thanks to their extensive ramified processes. Interestingly, in healthy retinas, processes are highly dynamic structures that show a high degree of movement, which is not followed by the movement of their soma (Lee et al., 2008). Following stress or retinal injury, microglial cells start to extend their processes toward the injured area. In addition, microglial morphologies change from arborized to ameboid shape. The morphological changes and soma migration toward the injured or stressed part of the retina are usually considered a clear sign of microglia activation (Lee et al., 2008; Cuenca et al., 2014; Di Marco et al., 2019; Rashid et al., 2019). For this reason, we considered microglia migration to the ONL and their changes in shape (evaluated with Scholl analysis) as markers for augmented reactivity following implant surgery. We counted, in all experimental groups, the number of IBA-1-positive cells in the ONL layer (Figure 9A). We observed an increased number of IBA-1 positive cells in the “implant” region of both sham and prosthesis eyes with respect to non-implanted controls (Figure 9B). However, the total number of microglia did not change in the different samples (Figure 9C). When we performed Scholl analysis to quantify the activation of microglia in terms of number and extension of processes, microglial activation was closely similar between the experimental groups, with no signs of the harmful reactive microglia in the implanted retinas (Figure 9D).

**FIGURE 10:**
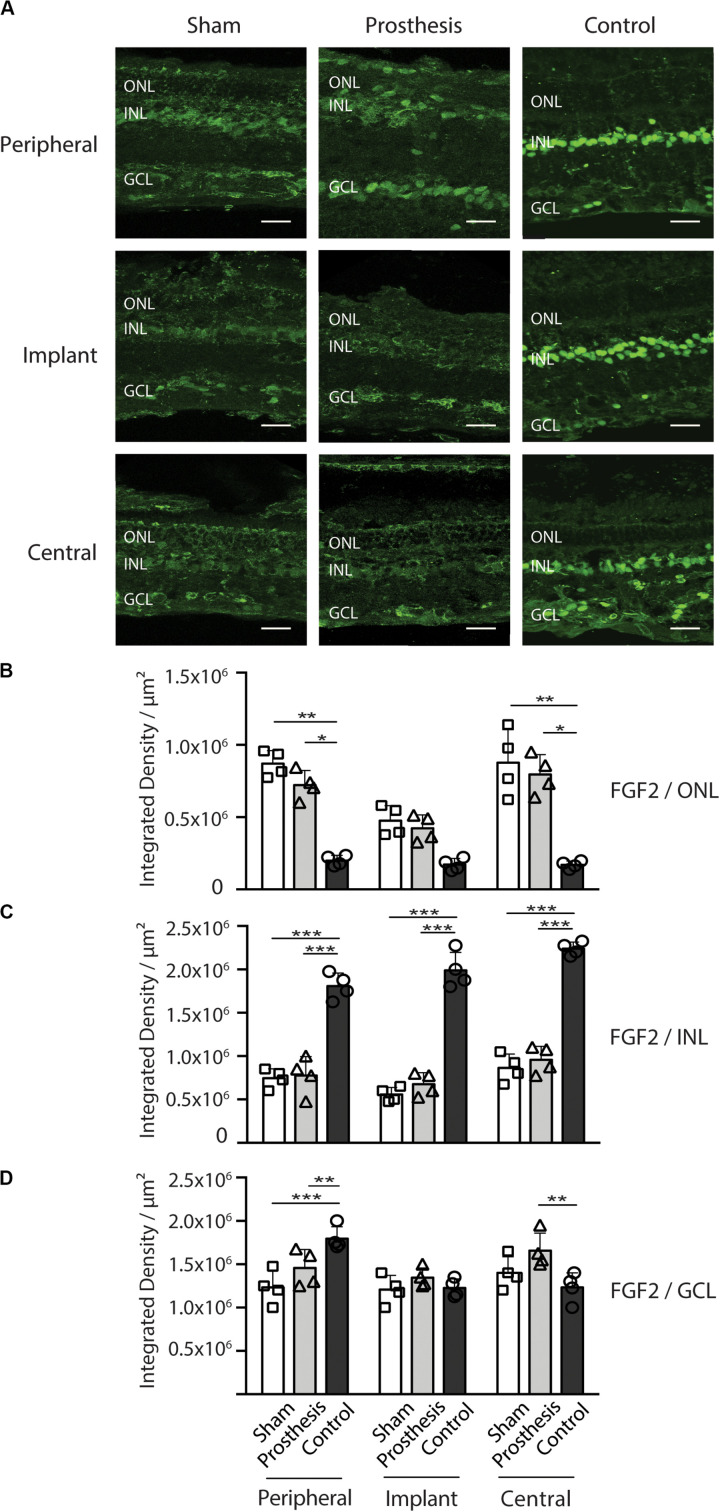
FGF2 expression in the retina after implantation of PET-based devices in the pig. **(A)** Representative retina sections stained with anti-FGF2 antibody (green). The immunoreactive areas were sampled as indicated in Figure 7B. ONL, outer nuclear layer; INL, inner nuclear layer; GCL, ganglion cell layer. Scale bars, 50 μm. **(B)** Bar plots showing the FGF2 levels in the ONL of sham-implanted (white), prosthesis-implanted (light gray), and non-implanted control (dark gray) groups measured in the peripheral, implant and central regions of the retina. In both the peripheral and central areas, prosthesis- and sham-implanted retinas are significantly more positive than the control retinas**. (C)** Bar plots showing the FGF2 levels in the INL of the same experimental groups shown in B. All areas of control retinas were significantly different from both sham- and prosthesis-implanted retinas. **(D)** Bar plots showing the FGF2 levels in the GCL of the same experimental groups shown in B. No variation of FGF2 immunoreactivity was observed between the implant area of both sham- and prosthesis-implanted eyes as compared to control eyes. A significant decrease of FGF2 expression was found in the peripheral area of both sham- and prosthesis-implanted eyes as compared to control eyes. In contrast, a significant increase of FGF2 was observed in the central area of prosthesis-implanted eyes with respect to controls. All data are means ± SD with superimposed experimental points. *p < 0.05; **p < 0.01; ***p < 0.001; two-way ANOVA/Tukey’s test (*n* = 4).

Muller cells constitutively synthesize FGF2 that, following retinal insults such as optic nerve sectioning or photoreceptor damage, translocates to photoreceptors. There, FGF2 binds to its specific receptor (FGFR-1), increasing photoreceptor stability, survival, and reducing photoreceptor synaptic function (Gargini et al., 2004; Valter et al., 2005; Hochmann et al., 2012). We measured FGF2 intensity in all layers of the retina (Figure 10A), with special reference to the ONL. We found an increase of FGF2 level in the “peripheral” and “central” ONL areas of sham- and prosthesis-implanted eyes and, to a lesser extent, in the “implant” area (Figure 10B). The lower FGF2 density seen in the “implant” area likely reflects the loss of photoreceptors. Both sham- and prosthesis-implanted eyes showed less fluorescence with respect to control retinas in the Muller cells soma (Figure 10C). The fluorescence levels in the GCL of the “central” region were higher in controls with respect to sham- and prosthesis-implanted retinas, slightly higher in the peripheral area of prosthesis-implanted retinas, and equivalent to controls in all the other regions (Figure 10D).

## Discussion

To evaluate the action of the polymeric prosthesis, successfully tested in the RCS rat, in the swine model, we used the bilateral subretinal implantation of the retinal prosthesis in naïve pigs as experimental paradigm. After initial unsuccessful attempts of retinal implantation by trans-scleral route, we decided to use an intravitreal approach. The prosthetic device initially used in rats (Maya-Vetencourt et al., 2017), consisted of 3 layers: P3HT, PEDOT:PSS, and crystallized silk fibroin as substrate. However, due to the intrinsic rigidity of the crystallized silk fibroin, we used the same starting material to ESF fabrics characterized by an extremely uniform surface, high flexibility and absence of memory after bending. The modification of the substrate was to facilitate the surgical implant procedure and, at the same time, to increase the area of retina coverage. Despite the technical success of the implant design, we found an undesired retinal reaction (PVR) to the ESF substrate, as assessed by indirect ophthalmoscopy and histology, associated with the complete disappearance of the ff-ERG b-wave signal in the implanted eyes. We then replaced the ESF with PET, while keeping the other layers of the prosthesis intact. To test the efficacy of this material, we performed control experiments in dystrophic RCS rats. In line with previous findings (Maya-Vetencourt et al., 2017), RCS dystrophic animals implanted with the PET-based prosthetic showed a significant rescue of both light sensitivity and visual acuity that was virtually absent from sham-implanted rats.

After testing the efficacy of the new prosthesis in rodents, we implanted subretinally the prosthesis or the substrate alone in pigs with normal vision. Following the surgical implant of the sham device, the ff-ERG b-wave amplitude was reduced with respect to the signal recorded before the implant, confirming the partial retinal dysfunction after surgery and testifying the degeneration of photoreceptors in the retinal area overlying the implant. Notably, the eyes that received the active prosthesis, equal in size as compared to the sham device and implanted with the same surgical procedure, displayed fully preserved ff-ERG b-wave amplitudes, in spite of the implant-induced local photoreceptor degeneration. This result indicates not only the absence of adverse inflammatory reactions to the implantation of the active device in the normal retina, but also a functional compensation of the induced photoreceptor degeneration carried out by the photosensitive prosthesis. Moreover, *SD*-OCT images revealed that the external retina remained in tight contact with the prosthetic, with only a limited reduction of the outer retina thickness in the “implant” area, but not in neighboring regions. These findings show that the optimized prosthetic does not induce severe tissue damage to outer layers of retina and that the surgical procedure for the subretinal prosthesis is well standardized.

Histological analyses revealed a moderate degree of stress in photoreceptors localized in the site of “implant,” in the absence of adverse effects on the retinal structure outside the implant area. In the retina, GFAP is constitutively expressed in astrocytes and Muller cells, and becomes upregulated under stress conditions (Lewis and Fisher, 2003; Edwards et al., 2016; Luna et al., 2016; Zhang et al., 2017; Lu et al., 2018). Interestingly, we observed no changes in GFAP expression after 10 days of surgery in both sham-implanted and prosthesis-implanted eyes as compared to non-implanted controls. Furthermore, microglia activation assessed by IBA-1 immunoreactivity was significantly enhanced with respect to controls only at the “implant” area of both sham-implanted and prosthesis-implanted eyes, but not in the neighboring areas of the retina. Additionally, Scholl analysis unveiled no differences in the morphology of microglial cells among the various experimental groups, indicating the absence of microglia activation. Our experimental findings point toward the retinal recovery from the surgical stress associated with the prosthetic implant and to an excellent biocompatibility of the PET substrate.

Another important aspect in the field of vision science research is the trophic effect induced by retinal surgery, which could *per se* enhance photoreceptors’ survival. To address this issue, we performed immunohistochemistry for FGF2, a trophic factor constitutively expressed in Muller cells and retinal ganglion cells (Walsh et al., 2001; Valter et al., 2002) whose upregulation and translocation to the ONL in the retina is a typical landmark of photoreceptor stress (Xiao et al., 1998, 1999; Sannita et al., 2004; Valter et al., 2005; Rutar et al., 2010; Hochmann et al., 2012). An induction of FGF2 levels, likely due to the surgical implantation procedure, was found in the neighboring areas of the retina. This finding, together with the fact that no functional recovery was seen in sham-implanted animals, confirms the previously described role of FGF2 in the retinal response after the surgery (Valter et al., 2005). Interestingly, no additional signs of retinal stress were detected. Neither an increase of GFAP-positive cells, nor a morphological activation of IBA-1-positive cells as assessed by Scholl analysis was observed in the area covered by the prosthesis. In humans, it was reported that ff-ERG suppression in the damaged retina correlates with the up-regulation of FGF2, but not with the extension of damaged area (Di Loreto et al., 2002). Notably, FGF2 upregulation has been found to be deleterious for visual responses to light in animal models (Gargini et al., 1999). The decrease of ff-ERG signals observed in animals sham-implanted with the PET-only devices indicates that the up-regulation of FGF2 levels in the “peripheral” and “central” areas of the retina 10 days after the implant reduced the retinal responses, by acting at the level of the first synapse photoreceptors-bipolar cell (Valter et al., 2002, 2005). On the other hand, the total preservation of visual responses after implantation of the fully active device, in spite of photoreceptor degeneration overlying the implant area, indicates that the polymers might compensate for this phenomenon by acting on second order neurons (bipolar cells) thus rendering the degenerated area of the retina light sensitive.

## Conclusion

Our findings obtained in a large animal model such as the biomedical pig highlight the possibility of developing a new generation of photosensitive conjugated polymer/PET-based prosthetic devices that are highly biocompatible and suitable for subretinal implantation in patients suffering from degenerative blindness.

## Data Availability Statement

The data that supports the plot within this paper together with other findings of this study are available from the corresponding author upon reasonable request.

## Ethics Statement

The animal study was reviewed and approved by the Italian Ministry of Health.

## Author Contributions

FaB, MB, GL, and GP supervised, coordinated, and financed all the activities on pigs and rats. JFM-V performed the *in vivo* electrophysiology experiments in rodents and followed the electroretinographic analyses on pigs under the supervision of FaB. SD and MD carried out the immunohistochemical and histological analysis under the supervision of SB. MM executed the optical coherence tomography. GP and MM developed and performed the subretinal implantation of the prosthesis. GM and AD fabricated the prostheses and characterized them under the supervision of GL. DV, AE, and FrB performed all the procedures on pigs including general husbandry, anesthesia, electrophysiological analyses, and final samplings under the supervision of MB. WS supported the electroretinographic analysis on pigs. JFM-V, SD, and FaB wrote the manuscript. All authors discussed the experimental results and commented on the script.

## Conflict of Interest

The P3HT material studied in this paper is subject of the US patent application US 16/005248 ‘Eye-injectable polymeric nanoparticles and method of use therefor’ by Istituto Italiano di Tecnologia and Ospedale Sacrocuore Don Calabria, with JM-V, MM, GP, FaB, and GL as inventors. The remaining authors declare that the research was conducted in the absence of any commercial or financial relationships that could be construed as a potential conflict of interest.

## Supplementary Material

Supplementary Figure 1 *SD*-OCT images of the eyes in the experimental pigs. **(A)** Representative *SD*-OCT scans of the left eyes in the G1 and G2 naïve animals, highlighting the optimal health state of retinal layers. **(B)** Representative images of the eyes of E2 and E3 pigs showing major morphological alterations in the external retinal layers after the ESF implant. **(C,D)** The implantation of the PET-based retinal prosthesis or PET alone (E31 and E34 pigs) causes no morphological alterations in external layers of the retina after the subretinal implant. Scale bars: 100 μm.
