# Supplementary figures and images for: Biocompatibility of a Conjugated Polymer Retinal Prosthesis in the Domestic Pig
Source: Front Bioeng Biotechnol. 2020 Oct 15;8:579141. doi: 10.3389/fbioe.2020.579141 (PMC7605258; doi:10.3389/fbioe.2020.579141)

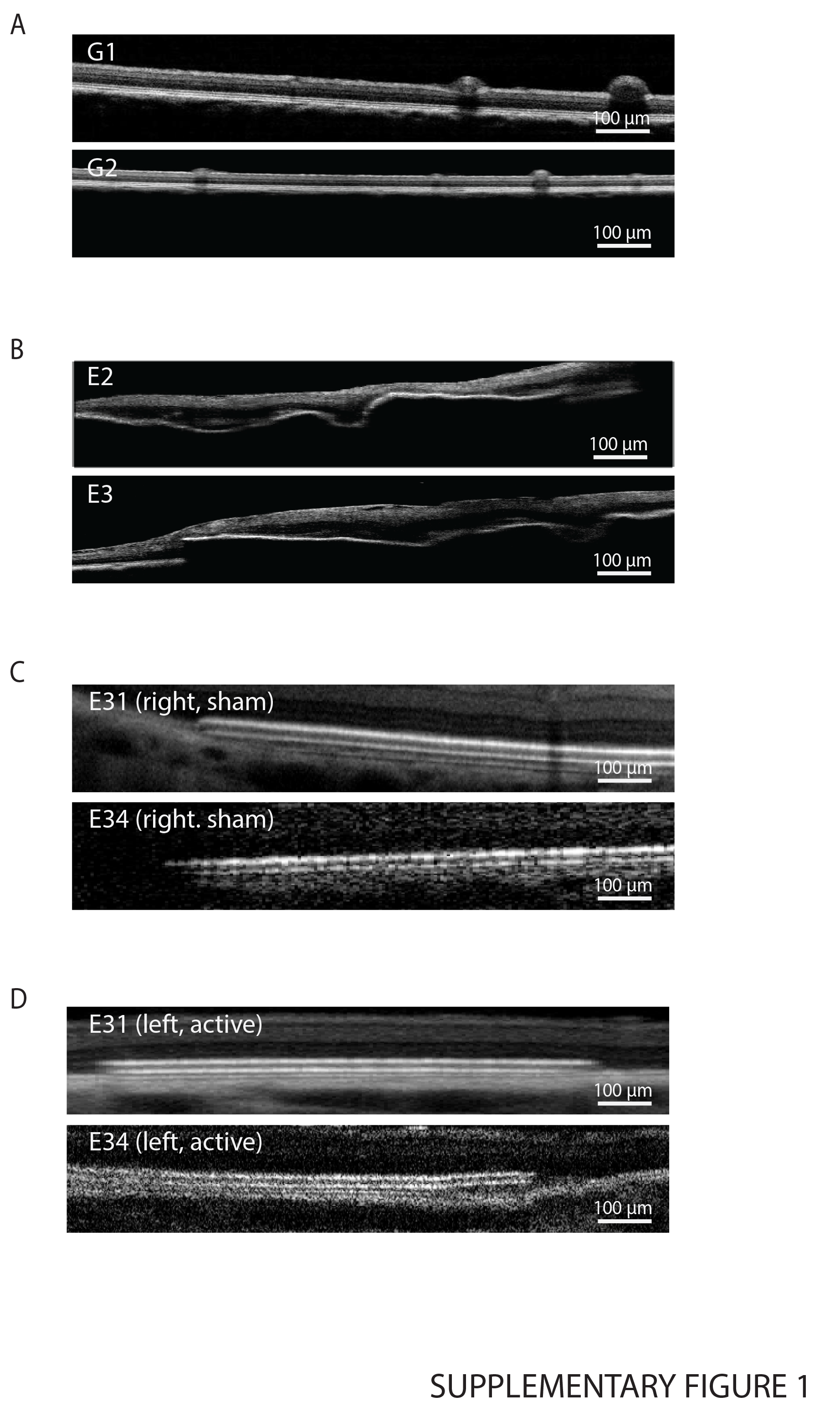

Supplement: Supplementary Figure 1 — SD-OCT images of the eyes in the experimental pigs. (A) Representative SD-OCT scans of the left eyes in the G1 and G2 naïve animals, highlighting the optimal health state of retinal layers. (B) Representative images of the eyes of E2 and E3 pigs showing major morphological alterations in the external retinal layers after the ESF implant. (C,D) The implantation of the PET-based retinal prosthesis or PET alone (E31 and E34 pigs) causes no morphological alterations in external layers of the retina after the subretinal implant. Scale bars: 100 μm. [file Image_1.TIF]
